# Distributions of trace elements with Long‐term grazing exclusion in a semi‐arid grassland of Inner Mongolia

**DOI:** 10.1002/ece3.70072

**Published:** 2024-08-13

**Authors:** Juan Hu, Daowei Zhou

**Affiliations:** ^1^ State Key Laboratory of Black Soils Conservation and Utilization, Northeast Institute of Geography and Agroecology Chinese Academy of Sciences Changchun China; ^2^ Jilin Provincial Key Laboratory of Grassland Farming Changchun China

**Keywords:** grassland, long‐term grazing exclusion, proportion, stock, trace element

## Abstract

Trace elements are the essential mineral nutrients in grassland, however, we still know little about the distributions of trace elements in grassland with long‐term grazing exclusion. The contents, stocks, and proportions of iron (Fe), aluminum (Al), manganese (Mn), and boron (B) in green plant‐litter‐root‐soil were evaluated by enclosing for 18, and 39 years inside the fence (F18 and F39) and grazing outside the fence (F0) in Inner Mongolia grassland. The results showed that F18 and F39 decreased the stocks of Fe, Al, and Mn in green plant and root compared to F0 (*p* < .05), while increased the stocks of them in litter (*p* < .05). The stock of Fe, Al, and Mn in green plant at F39 was 28.6%, 13.9%, and 39.2% higher than that at F18. The stocks of four trace elements in first layer litter at F39 were increased by 12.7%–52.2% compared to F18, whereas the stocks of them in third layer litter were decreased by 32.2%–42.5%. The F18 obviously increased the stocks of Fe and Mn in soil, especially B (*p* < .05). While the stocks of these trace elements in soil at F39 were 9.1%–28.0% lower than that at F18, especially B (*p* < .05). In conclusion, the trace elements were mainly shifted from green plant and root to soil and third layer litter with 18‐year grazing exclusion. Compared to 18‐year grazing exclusion, the trace elements were shifted from third layer litter and soil to root with 39‐year grazing exclusion.

## Graphical abstract

The Fe toxicity occurred in the grassland with long‐term grazing, and Mn deficiency was observed in the grassland with long‐term grazing exclusion. The trace elements were mainly shifted from the green plant and root to soil and litter with 18‐year grazing exclusion compared to long‐term grazing. The trace elements were mainly shifted from soil and litter to root with continue 39‐year grazing exclusion compared to grazing exclusion for 18 years.

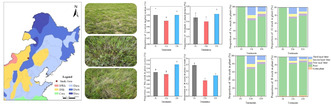

## 1 INTRODUCTION

Grasslands constitute about 40% of the Earth's land surface and provide habitats for biodiversity and important ecosystem services (Stromberg & Staver, 2022). Inner Mongolia grassland is one of the most important temperate grasslands, and the livestock husbandry is the preponderant industry in this region. However, 70% of grassland has been degraded because of the factors of nature and human (Bai et al., 2015; Deng et al., 2014; Hafner et al., 2012; Lin et al., 2015; Liu et al., 2019; Piñeiro et al., 2009; Wang et al., 2017; Yan et al., 2009). Grassland degradation seriously affects the yield and quality of pasture (Zhou et al., 2011), and hence threatens the livestock husbandry (Kang et al., 2007). Grazing exclusion has been widely regarded as an effective management strategy for restoring degraded grassland around the world (Golodets et al., 2010; Hu et al., 2016; Javier et al., 2016; Roy et al., 2023; Shrestha & Stahl, 2008; Wu et al., 2010).

Trace elements are the essential mineral nutrients in the grassland ecosystem (Hansch & Mendel, 2009; Yadav, 2010), although their contents only accounted for 1% of the dry matter of pasture. The lack or excess of trace elements is an important factor that restricts the yield and quality of pasture and the health of animal, which causes enormous economic losses to the herders (Ning et al., 2022; Shen et al., 2006; Xin et al., 2011). Therefore, the research on the cycle of trace elements can help us maintain the community structure and ecosystem function of grassland, as well as better understand the deficiency and toxicity of trace elements in pasture (Ågren, 2008). Grazing exclusion can alter the trace elements in soil and change the contents of these elements in pasture (Han et al., 2011; Liu et al., 2014; Oliveira Filho et al., 2019). Unfortunately, we do not know much about the distributions of trace elements in grassland with long‐term grazing exclusion.

Studies have found that long‐term grazing exclusion can promote the restoration of vegetation and mineral nutrients in plant or soil in degraded grasslands (Congio et al., 2021; Hu et al., 2020; Liu et al., 2017; Wang et al., 2018). However, long‐term grazing exclusion has resulted in excessive accumulation of litter on the surface of soil (Hou et al., 2019; Wang et al., 2017). Litter is the vegetation that cannot conduct photosynthesis. Its quantity is related to the biomass of green plants (Hou et al., 2019; Wang et al., 2017). The decomposition of litter is of great significance to the nutrient return from plant to soil in grassland (Ball et al., 2014; Wang et al., 2011). It is mainly depended on the soil properties and plant characteristics (Naeem et al., 2021; Song et al., 2017). The high soil moisture induced by large litter cover can also improve the nutrient availability of soil (Deutsch et al., 2010). Moreover, the root biomass of grasslands is 2 to 30 times higher than the above‐ground standing biomass (Chen et al., 2006; Naeem et al., 2021). The variations of productivity and composition of above‐ground community and soil physical–chemical properties in grassland are vital for the allocations of photosynthetic products between above‐ground and below‐ground (Gao et al., 2008). In sum, green plant, litter, root, and soil in grassland should be viewed as an entirety to deep understand the nutrient cycle of grassland. However, there is less information for the interrelationship of nutrients in the green plant‐litter‐root‐soil system in the long‐term grazing exclusion grassland, especially trace elements.

Therefore, the objectives of this study are as follows: (1) exploring the distributions of Fe, Al, Mn, and B in green plant‐litter‐root‐soil in grassland with long‐term grazing and with 18‐ and 39‐year grazing exclusion; (2) revealing the sensitivity and response of trace elements to long‐term grazing or long‐term grazing exclusion. We hypothesize that proportions of trace elements in green plant‐litter‐root‐soil would be altered with different years of long‐term grazing exclusion. This study will provide a scientific basis for the restoration of degraded grasslands, and the study of trace elements in semi‐arid grasslands of Inner Mongolia.

## 2 METHODOLOGY

### 2.1 Study site

**FIGURE 1:**
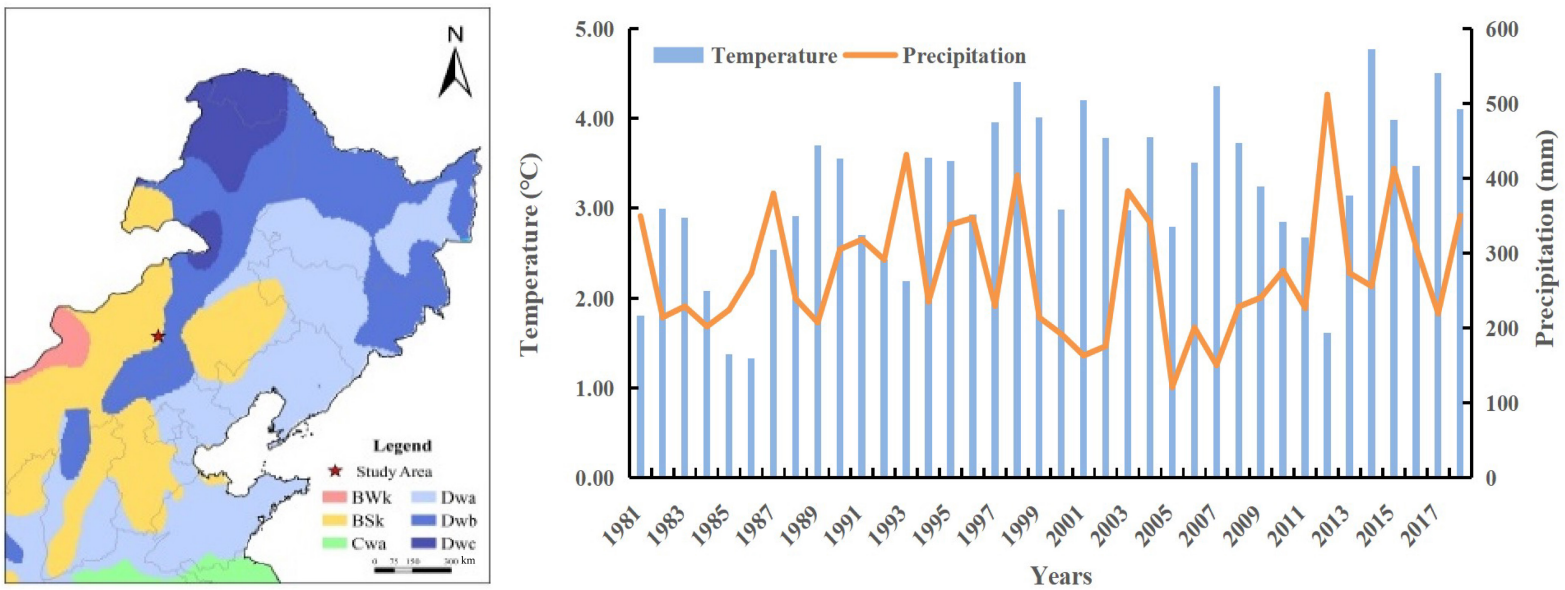
The map of research region and the mean temperature and mean precipitation from 1981 to 2018 year in Xilin River Basin.

This study was conducted at the Inner Mongolia Grassland Ecosystem Research Station of the Chinese Academy of Sciences (IMGERS, 43°38′ N, 116°42′ E), which is located in Xilin River Basin of Inner Mongolia, China. The region has a semi‐arid grassland climate with the annual average temperature of 2.3°C. The annual average precipitation is 330 mm. The annual evaporation is 4~5 times of the precipitation. The soil is described as a dark chestnut, with a loamy sand texture (Bai et al., 2015). The original forage species were *Stipa grandis* and *Leymus chinensis*, and the mean percentage of *Stipa grandis* and *Leymus chinensis* was 57% and 21%, respectively. Strong winds occur from March to May, and the average monthly speed can reach 4.9 m/s. Wind erosion and dust storms are the common phenomena in this region (Hoffmann et al., 2008). The map of research region and the mean temperature and mean precipitation in research region are shown in Figure 1 (Hu et al., 2021).

### 2.2 Experimental design and sampling

**FIGURE 2:**
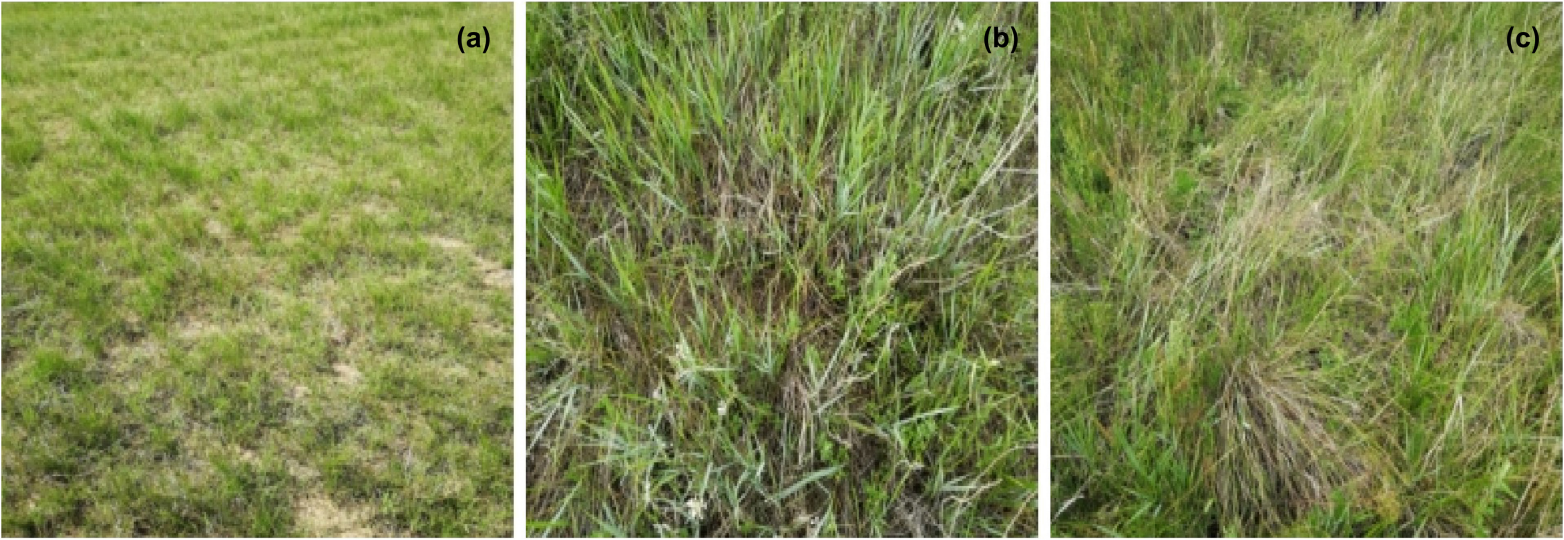
Grazing plot (a), enclosing 18 years plot (b), and enclosing 39 years plot (c).

The experiment was conducted on August 2019. The experimental sites were composed of three plots, including the 39‐year grazing plot outside of fence (F0), the 18‐year grazing exclusion plot inside of fence (F18, grazing exclusion from 2001) and the 39‐year grazing exclusion plot inside of fence (F39, grazing exclusion from 1980) (Figure 2). The grazing intensity was 5 sheep/hm^2^/year in the long‐term grazing plot. A typical transect (100 m long) was randomly located in each plot. Ten quadrats were established at 10 m intervals in each transect for vegetation and soil sampling within each transect.

Green plant, litter, root, and soil samples were collected in the same quadrat of 1 × 1 m. Green plant and litter were collected and weighed after dried in 105°C in the laboratory. Litter was divided into first layer litter (standing litter), second layer litter (falling litter), and third layer litter (fragmented litter). Firstly, we collected the standing litter in each quadrat. Secondly, we collected the green plant in each quadrat. Thirdly, we collected the falling litter on the surface soil, and then collected the fragmented litter on the surface soil.

Ten soil sampling locations were evenly spaced and treated as 10 replications within each transect. From each soil sampling location, three soil cores were collected to make a composite sample in each soil layer (0–10, 10–20, 20–30, 30–50, 50–70, and 70–100 cm depth). Soil samples were placed into plastic bags and then air‐dried in the laboratory. Then, the soils were sieved through a 2 mm sieve and used for determination.

The root samples were collected using the method of soil auger. One soil core was collected (0–10, 10–20, 20–30, 30–50, 50–70, 70–100 cm depth) from each soil sampling location. The 0–10 and 10–20 cm samples were mixed as 0–20 cm root sample, and 20–30 and 30–50 cm samples were mixed as 20–50 cm root sample, and 50–70 and 70–100 cm samples were mixed as 70–100 cm root sample. The root samples were packed into mesh bag and were washed by deionized water. Then root were collected after dried in 105°C.

The Fe, Al, Mn, and B contents in plant and soil were determined using a flame atomic absorption machine (Varian Model AA240).

### 2.3 Statistical analysis

All data were statistically analyzed using a 10.0 SPSS software. We calculated the standard errors and compared the means of parameters among different treatments through one‐way analysis of variance (ANOVA). The least significant difference (LSD) test was performed. Pearson correlation was used to assess the relationships of trace elements contents among green plant, litter, root, and soil.

The equation of stock of mineral element is:

$$\begin{array}{c} \text{Mineral element stock in soil}\;\left(\frac{g}{m^{2}}\right)=\text{mineral element content}\;\left(\frac{g}{\mathrm{kg}}\right)\\ \;\times \text{bulk density}\left(\frac{g}{{\mathrm{cm}}^{3}}\right)\times \text{soil deep}\;\left(\mathrm{cm}\right)\times 10,000. \end{array}$$

Mineral element stock in plantgreen plant,litter,and rootgm2=mineral element contentgkg×drymatter of plantgm2×10−3.

## 3 RESULTS

### 3.1 Contents of trace elements in grasslands

The content of Fe, Al, Mn, and B in green plant at F18 was 86.2%, 79.5%, 63.0%, and 41.2% lower than that at F39, respectively (*p* < .05). F39 greatly increased the B content in green plant compared to F18 (*p* < .05; Table 1).

**TABLE 1:** The contents of trace elements in grassland system after long‐term grazing exclusion (Mean ± standard error).

	Fe (g/kg)			Al (g/kg)			Mn (mg/kg)			B (mg/kg)		
	F0	F18	F39	F0	F18	F39	F0	F18	F39	F0	F18	F39
Green plant	1.30 ± 0.3^a^	0.18 ± 0.0^b^	0.20 ± 0.0^b^	1.22 ± 0.3^a^	0.25 ± 0.1^b^	0.21 ± 0.0^b^	76.1 ± 10.3^a^	28.1 ± 0.8^b^	37.8 ± 4.9^b^	6.6 ± 0.8^a^	3.9 ± 0.5^b^	7.9 ± 1.3^a^
Litter												
First layer	3.85 ± 1.5^a^	4.22 ± 0.6^a^	5.49 ± 2.5^a^	3.84 ± 1.5^a^	4.82 ± 0.8^a^	5.41 ± 2.4^a^	127.3 ± 37.0^a^	112.4 ± 26.4^a^	138.4 ± 66.6^a^	7.1 ± 0.4^a^	6.0 ± 1.5^a^	5.8 ± 1.9^a^
Second layer		6.56 ± 0.8	7.24 ± 2.4		7.42 ± 0.7	8.21 ± 2.5		186.3 ± 23.8	199.0 ± 57.4		8.6 ± 1.2	7.2 ± 1.5
Third layer		11.94 ± 2.4	12.00 ± 0.7		14.39 ± 0.4	13.23 ± 0.7		385.5 ± 42.1	343.0 ± 9.8		13.8 ± 0.9	10.8 ± 0.3
Root												
0–20 cm	6.97 ± 2.1^a^	4.25 ± 0.2^a^	5.75 ± 0.6^a^	9.31 ± 2.4^a^	6.35 ± 0.3^a^	7.59 ± 0.8^a^	191.4 ± 16.3^a^	158.8 ± 17.6^a^	225.4 ± 46.7^a^	9.0 ± 0.7^a^	7.8 ± 0.6^a^	9.0 ± 0.7^a^
20–50 cm	5.20 ± 0.5^a^	3.57 ± 0.3^a^	3.89 ± 0.4^a^	7.83 ± 0.9^a^	5.40 ± 0.5^b^	5.11 ± 0.4^b^	121.8 ± 13.3^a^	89.5 ± 3.4^a^	102.5 ± 16.3^a^	12.9 ± 0.5^a^	10.1 ± 1.7^a^	9.0 ± 0.8^a^
50–100 cm	4.93 ± 0.5^a^	3.47 ± 0.2^b^	3.94 ± 0.1^ab^	7.28 ± 0.6^a^	5.06 ± 0.1^b^	5.23 ± 0.1^b^	113.8 ± 3.2^a^	88.4 ± 8.4^a^	107.0 ± 13.4^a^	12.8 ± 0.2^a^	13.9 ± 0.2^a^	9.9 ± 1.8^b^
Soil												
0–10 cm	14.89 ± 0.2^a^	16.62 ± 1.4^a^	16.77 ± 0.3^a^	26.20 ± 1.5^a^	24.49 ± 1.3^a^	23.40 ± 0.8^a^	319.5 ± 5.1^a^	338.4 ± 8.5^a^	324.9 ± 8.9^a^	7.4 ± 0.4^a^	12.6 ± 3.0^a^	11.3 ± 0.8^a^
10–20 cm	14.36 ± 0.3^a^	14.88 ± 0.4^a^	14.52 ± 0.5^a^	26.02 ± 2.3^a^	25.07 ± 2.9^a^	20.64 ± 1.0^a^	303.0 ± 10.4^a^	312.0 ± 3.3^a^	280.9 ± 15.4^a^	6.1 ± 0.6^a^	9.2 ± 1.7^a^	8.1 ± 0.9^a^
20–30 cm	13.36 ± 0.3^a^	14.34 ± 0.5^a^	13.52 ± 0.2^a^	26.17 ± 2.7^a^	20.87 ± 2.2^a^	22.25 ± 2.0^a^	288.9 ± 20.1^a^	274.2 ± 11.0^a^	288.2 ± 13.3^a^	4.6 ± 0.3^a^	8.2 ± 2.1^a^	7.2 ± 0.4^a^
30–50 cm	12.31 ± 0.4^a^	14.15 ± 1.0^a^	13.67 ± 0.2^a^	25.59 ± 2.8^a^	21.50 ± 1.8^a^	21.98 ± 1.8^a^	262.3 ± 10.0^a^	273.3 ± 16.1^a^	269.5 ± 6.4^a^	3.9 ± 0.7^b^	8.8 ± 2.2^a^	7.3 ± 0.1^a^
50–70 cm	13.07 ± 0.4^a^	14.15 ± 0.7^a^	13.36 ± 0.1^a^	27.89 ± 2.4^a^	25.14 ± 0.8^a^	20.45 ± 2.1^b^	288.8 ± 14.8^a^	308.4 ± 16.3^a^	277.8 ± 20.2^a^	5.0 ± 1.1^a^	8.4 ± 2.2^a^	6.3 ± 0.9^a^
70–100 cm	13.58 ± 0.2^a^	15.25 ± 0.3^a^	13.34 ± 0.1^b^	28.52 ± 0.9^a^	23.95 ± 1.4^b^	20.67 ± 0.3^b^	308.0 ± 11.7^a^	326.0 ± 17.3^a^	272.0 ± 4.5^b^	5.0 ± 0.3^b^	10.7 ± 1.2^a^	6.0 ± 0.7^b^

*Note*: Different lowercase letter in row indicates significant difference at .05 level among treatments (ANOVA).

The contents of trace elements in litter showed L3 > L2 > L1. The Fe and Al content of L1 at F18 was 9.6% and 25.5% higher than that at F0, respectively, whereas the Mn and B content was 11.7% and 15.5% lower than that at F0, respectively. The Fe, Al, and Mn content of L1 at F39 was 30.1%, 12.2%, and 23.1% higher than that at F18, respectively. The Fe, Al, and Mn content of L2 at F39 was 10.4%, 10.6%, and 6.8% higher than that at F18, respectively. The B content of L2 at F39 was 16.3% less than that at F18. The trace elements contents of L3 at F39 were slightly lower than that at F18 (Table 1).

The Fe, Al, and Mn contents in the root in 0–20 cm were higher than that of 20–50 and 50–100 cm. However, B content in the root in 0–20 cm was less than that of 20–50 and 50–100 cm. The Fe, Al, Mn, and B content of root in 0–20 cm at F18 and was 39.0%, 31.8%, 17.0%, and 13.3% less than that at F0. The Fe, Al, Mn, and B content of the root in 0–20 cm soil at F39 was 35.3%, 19.5%, 41.9% and 15.4% higher than that at F18, respectively (Table 1).

The contents of Fe and B in each soil layer at F18 were increased, whereas the contents of Al were decreased. The content of Fe and B in 0–100 cm soil at F18 was 9.6% and 81.1% higher than that at F0, respectively. However, the content of Al was 12.1% less than that at F0. The Fe, Al, Mn, and B content in 0–100 cm soil at F39 was 4.7%, 8.3%, 6.5%, and 20.1% less than that at F18, respectively (Table 1).

### 3.2 Stocks of trace elements in grasslands

#### 3.2.1 Trace elements stocks of green plant

**FIGURE 3:**
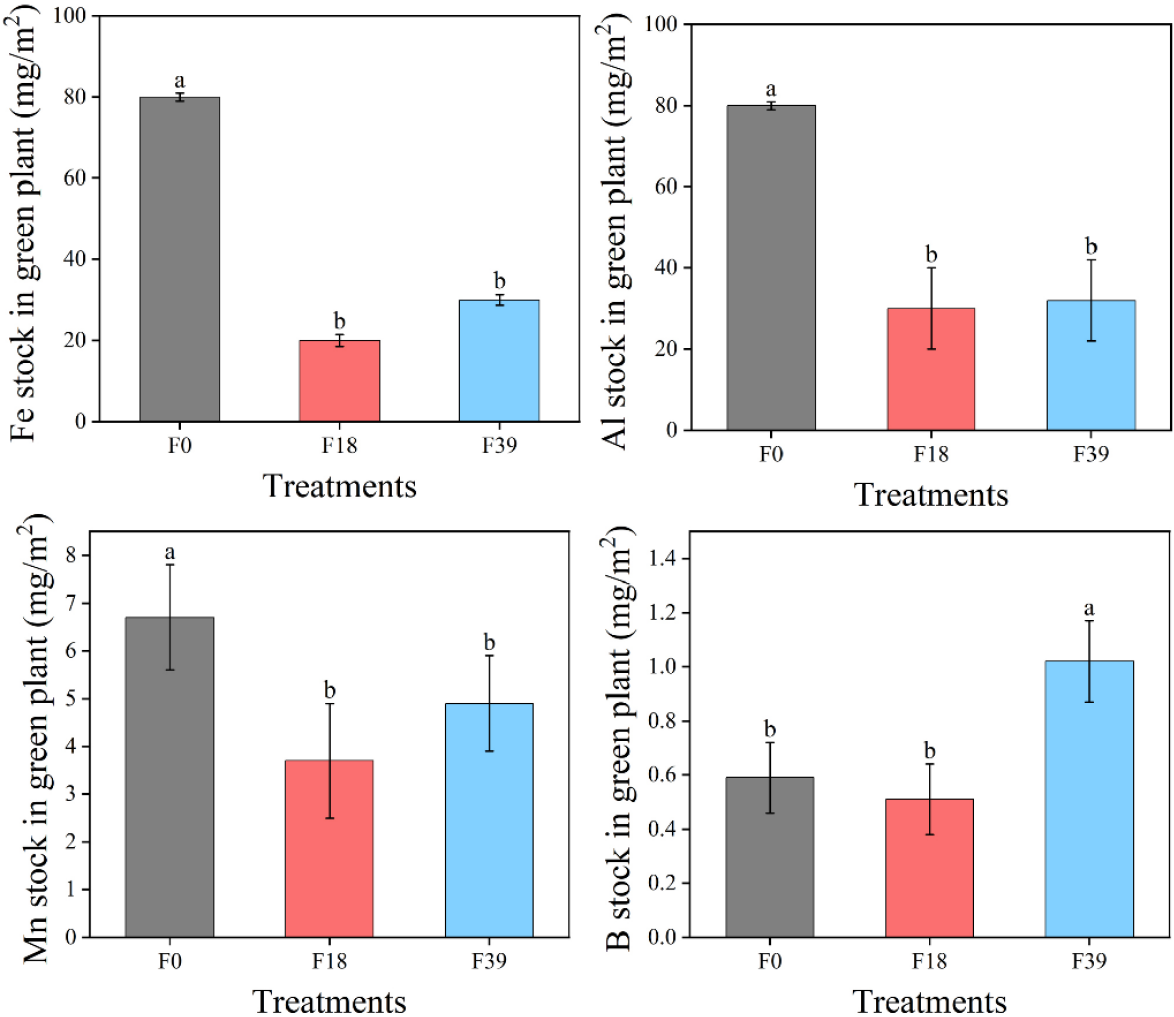
The Fe, Al, Mn, and B stocks of green plant after long‐term grazing exclusion. Lowercase letter indicated significant difference at .05 level (ANOVA). Error bars were one standard deviation.

The stock of Fe, Al, Mn, and B in green plant at F18 was 70.8%, 59.4%, 45.1%, and 12.9% lower than that at F0, respectively (*p* < .05). The B stock in green plant at F39 was 100.1% higher than that at F18 (*p* < .05; Figure 3).

#### 3.2.2 Trace elements stocks of root

**FIGURE 4:**
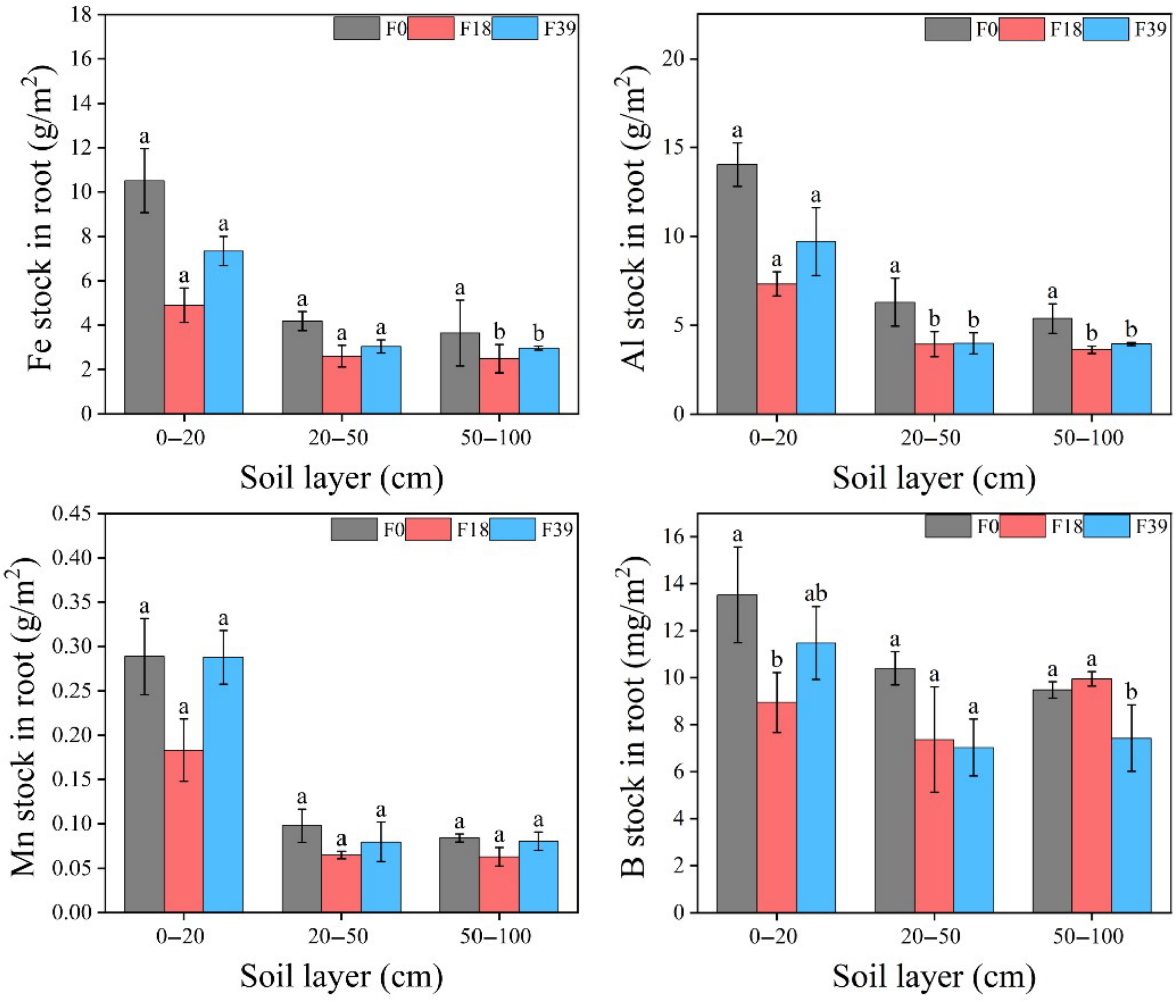
The Fe, Al, Mn, and B stocks of the root after long‐term grazing exclusion. Lowercase letter indicated significant difference at .05 level (ANOVA). Error bars were one standard deviation.

The Fe, Al, and Mn mainly stocked in 0–20 cm root, accounting for 34.1%–64.2% of 0–100 cm root. The Fe, Al, Mn, and B stock in 0–20 cm root at F18 was 53.4%, 47.8%, 36.6%, and 33.9% lower than that at F0, respectively. The Fe, Al, Mn, and B stock in 0–20 cm root at F39 was 50.1%, 32.4%, 57.2%, and 28.3% higher than that at F18, respectively (Figure 4).

#### 3.2.3 Trace elements stocks of litter

**FIGURE 5:**
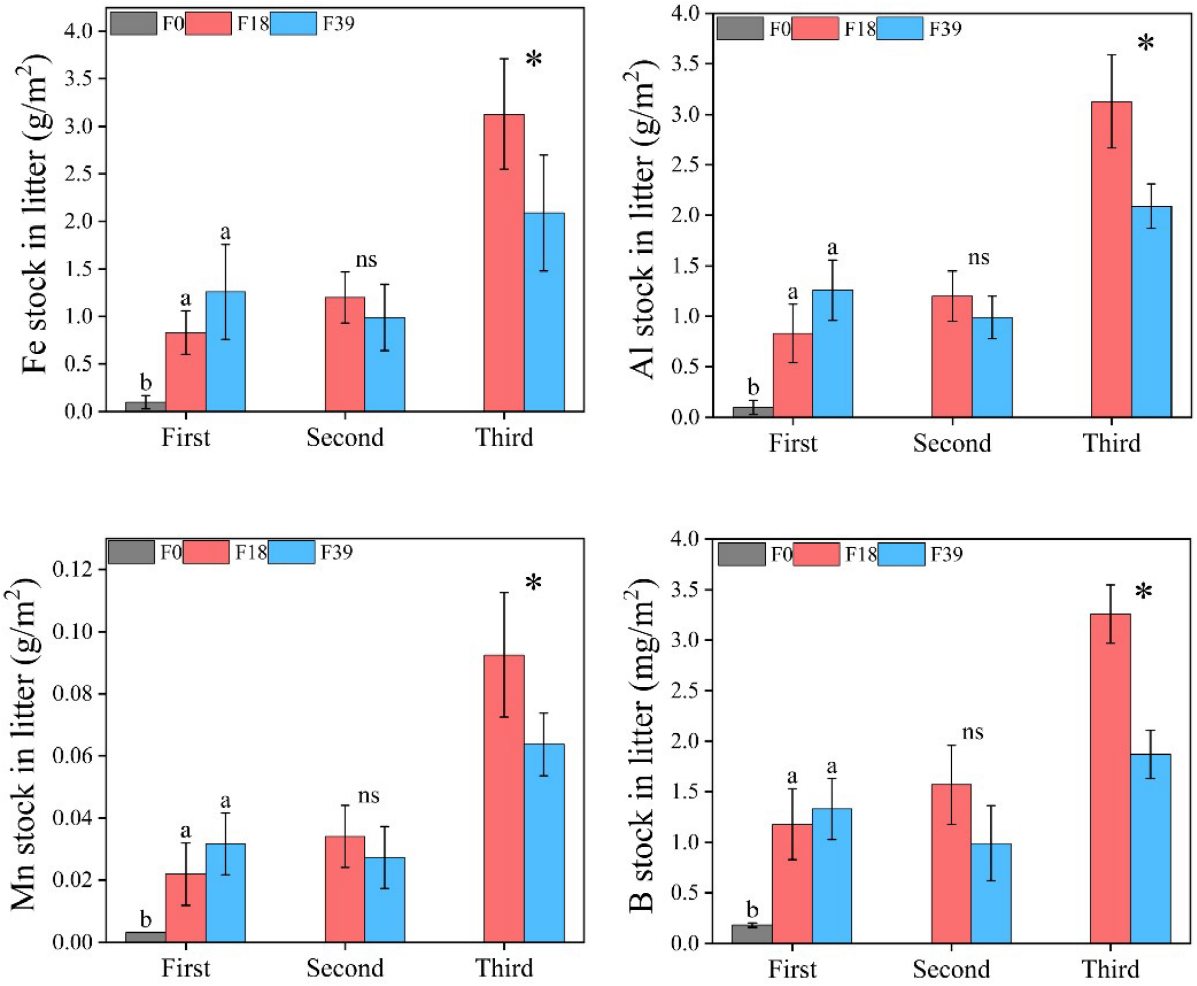
The Fe, Al, Mn, and B stocks in litter after long‐term grazing exclusion. Lowercase letter indicated significant difference at .05 level (ANOVA). *p < .05; **p < .01; ns, no significance. Error bars were one standard deviation.

The stock of Fe, Al, Mn, and B in total litter at F18 was 52.6, 46.3, 58.2, and 33.5 times higher than that at F0. The Fe, Al, Mn, and B stock in L3 at F39 was 33.2%, 32.2%, 36.2% and 42.5% less than that at F18 (*p* < .05; Figure 5).

#### 3.2.4 Trace elements stocks of soil

**FIGURE 6:**
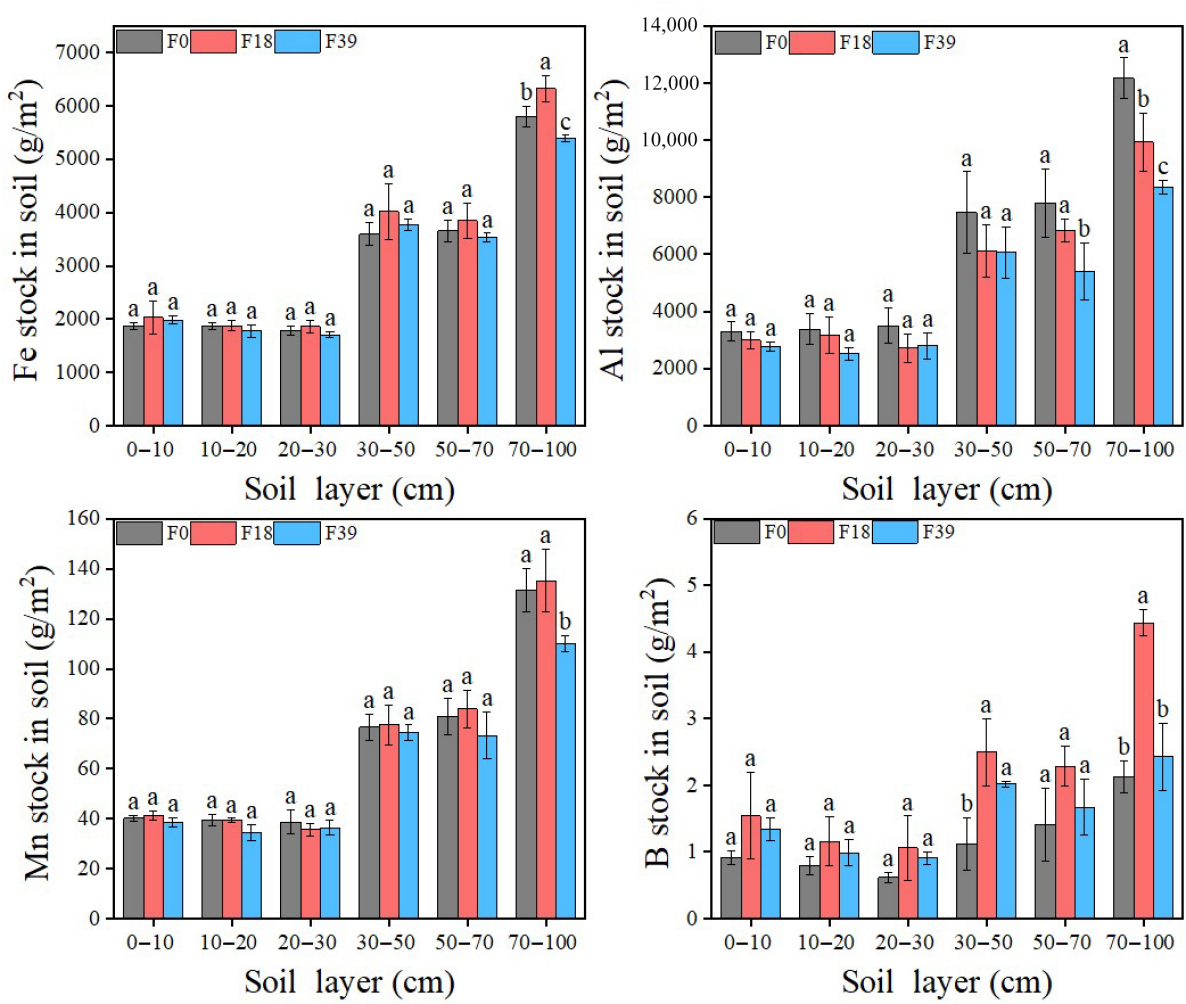
The Fe, Al, Mn, and B stocks in soil after long‐term grazing exclusion. Lowercase letter indicated significant difference at .05 level (ANOVA). Error bars were one standard deviation.

The Fe stock in 70–100 cm soil at F18 was 9.1% higher than that at F0 (*p* < .05). The B stock in 30–50 cm soil and 70–100 cm soil at F18 was 122.8% and 108.6% higher than that at F0 (*p* < .05). The B and Mn stock in 70–100 cm soil at F39 was 45.2% and 18.8% less than that at F18, respectively (*p* < .05). The Al stock in 70–100 cm soil at F18 was 18.4% less than that at F0. The Al stock in 50–70 cm soil and 70–100 cm soil at F39 was 21.1% and 16.0% less than that at F18, respectively (Figure 6).

### 3.3 Proportions of trace elements stocks

**FIGURE 7:**
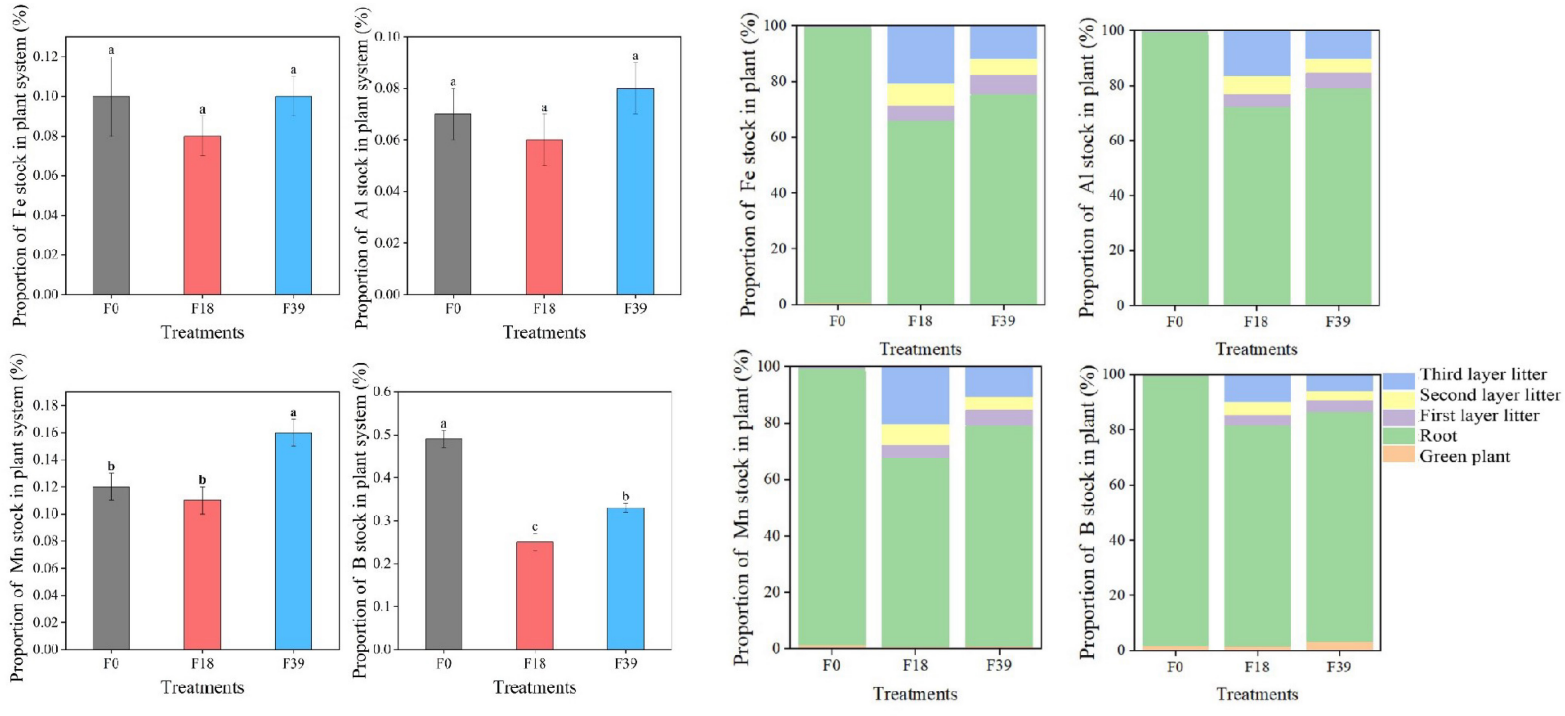
The proportions of trace elements stocks in plant system after long‐term grazing exclusion. Different lowercase letter indicates significant difference at .05 level among treatments (ANOVA).

The proportions of trace elements stocks in the plant system at F18 were less than that at F0. The B stock in plant system at F18 was 49.0% lower than that at F0 (*p* < .05). However, the proportions of trace elements stocks in the plant system at F39 were higher than that at F18. The Mn and B stock in F39 was 45.5% and 32.0% higher than that at F18, respectively (*p* < .05) (Figure 7).

Compared with that at F0, the proportions of trace elements stocks in litter at F18 were increased (especially in third layer litter) in the plant system, and the proportions of them in the root were decreased. Compared with that at F18, the proportions of trace elements stocks in litter at F39 were decreased from 18.31%–34.03% to 13.45%–24.50%, and the proportions of them stocks in the root were increased from 65.81%–80.13% to 75.35%–83.27%. After long‐term grazing exclusion, the proportion of Fe stock in litter was the highest, and the proportion of B stocks in green plant and root were greatest (Figure 7).

## 4 DISCUSSION

### 4.1 The trace elements shift from the plant via litter to soil during grazing exclusion 0 to 18 year

The study found that the proportions of trace elements stocks in soil with 18‐year grazing exclusion were increased relative to grazing, which suggested that the trace elements might shift from plant to soil. In plant system, we found that 18‐year grazing exclusion decreased the proportions of trace elements in green plant and root, whereas greatly increased them in litter. This indicated that the trace elements might shift from the plant via litter to soil during grazing exclusion 0 to 18 year. These variations of distributions of trace elements in plant‐litter‐soil might because of the variations of vegetation productivity (Cheng et al., 2016; Mekuria et al., 2007; Qasim et al., 2017), soil physical–chemical properties (Zeng et al., 2017), and mineral nutrients of soil (Dai et al., 2021; Hu et al., 2020). Long‐term grazing exclusion could increase the biomass of green plant owing to the reduction of livestock consumption by grazing animal, and the improvement of water holding capacity of soil by high plant coverage and low soil bulk density. The large amount of plant in long‐term grazing exclusion plot caused great litter accumulation. In further, the decomposition of litter caused the increase of trace elements stocks in soil.

The stocks of Fe, Al, and Mn in green plant were significantly decreased with 18 year grazing exclusion because of the great reduction of trace elements contents. This might be attributed to a dilution effect of largely increased vegetation productivity. Ning et al. (2022) reported that the low Fe and Mn contents in plants might resulted by their relative low bioavailability and the element dilution effect of the high biomass. The increase of green plant biomass of long‐term grazing exclusion was directly related to the decreased livestock consumption by grazing animals (Alberti et al., 2017; Steffens et al., 2008), as well as the improvement of soil physical–chemical properties and soil nutrients. The growth of the root was depended by variations of productivity and compositions of above‐ground community and soil physical–chemical properties. The stocks of Fe, Al, Mn, and B in the root with 18‐year grazing exclusion were decreased because of the reductions of root biomass and trace elements contents. Long‐term grazing exclusion can inhibit photosynthesis of plant because of the light restriction caused by the excess litter (Liu et al., 2019; Zhang et al., 2019). Therefore, this can reduce the allocation of photosynthetic product into the root, leading to an increase in the root mortality (Chen et al., 2006). Litter decomposition is the main way of nutrient return from plant to soil. It is of great importance for maintaining the balance of ecosystem (Ball et al., 2014; Ren et al., 2016). The study showed that the stocks of Fe, Al, Mn, and B in litter were significantly increased with 18‐year grazing exclusion. This attributed to the increases of litter biomass and trace elements contents. The contents of Fe and Al in first layer litter were increased with 18‐year grazing exclusion, whereas the contents of Mn and B in first layer litter were decreased. This attributed to the higher mobility in phloem of Mn and B (Zhao et al., 2004). The above results indicated that most trace elements were accumulated in litter from plant, especially from root. Further, the contents of trace elements in soil were mainly depended on the litter decomposition. The study showed that the stocks of Fe, Mn, and B in 0–100 cm soil were increased with 18‐ year grazing exclusion, whereas the stock of Al was obviously decreased. This was mainly because of the contents of trace elements in soil. The content of Al^3+^ in soil in a semi‐arid region in Brazil was decreased with grazing exclusion (Oliveira Filho et al., 2019). The Fe and Mn contents in soil were increased in the warm steppe with 29‐year grazing exclusion and in the alpine meadow steppe with 24‐year grazing exclusion (Li et al., 2011). The contents of Fe, B and Mn in the same region of this study were increased with 13‐year grazing exclusion. The plant coverage and litter accumulation on the soil surface were increased by long‐term grazing exclusion. This reduced the risks of soil nutrients loss caused by wind and water erosion (García et al., 2013; Hoffmann et al., 2008). Many studies found that the pH value was significantly decreased with long‐term grazing exclusion (Bai et al., 2021; Pei et al., 2008). This resulted in the increase in rock weathering and the release of trace elements into soil (Bowman et al., 2008). B content is very important for plant growth (Mills et al., 2023). Among these four trace elements, the B content in soil with long‐term grazing exclusion was increased most. The large amounts of B in soil were shifted from the root and litter because of the high mobility in phloem of B. However, the study found that the Al content in soil was decreased with long‐term grazing exclusion. This showed that most Al originated from green plant and root was accumulated in litter, and was difficultly to return into soil due to the poor mobility in phloem.

### 4.2 The trace elements shift from the litter via soil to plant during grazing exclusion 18 to 39 year

Continue grazing exclusion for 39 years obviously increased the biomass of root and first layer litter, while significantly decreased the biomass of second and third layers litter compared to 18‐year grazing exclusion. Therefore, continue grazing exclusion for 39 years further altered the distributions of trace elements in plant‐root‐litter‐soil relative to 18‐year grazing exclusion. The study found that grazing exclusion for 39 years decreased the proportions of trace elements stocks in soil compared with 18‐year grazing exclusion, which suggested that the trace element shifted from soil to plant. In plant system, we found that 39‐year grazing exclusion obviously decreased the proportions of trace elements in litter, whereas increased them in root. This indicated that the trace elements might shift from the litter via soil to plant during grazing exclusion 18 to 39 year. The decreases of trace elements stocks in litter were mainly resulted from the reductions of second and third layers litter biomass after continue grazing exclusion for 39 years. In the course of time, continue grazing exclusion for 39 years was in favor of the litter decomposition, and promoted more elements into soil for absorption by root.

The Fe and Mn contents in 0–40 cm soil in grassland with 33‐year grazing exclusion were less than that with 13‐year grazing exclusion in the same region of this study. We also found that the stocks and contents of Fe, Al, Mn, and B in 0–100 cm soil with 39‐year grazing exclusion were significantly less than that with 18‐year grazing exclusion. The Fe and Mn contents and stocks in the root were significantly increased with 39‐year grazing exclusion, especially in 0–20 cm soil. This might be attributed to the optical compensation effect of restoring the above‐ground plants. Large amounts of first layer litter might inhibit the establishment of seedling and restrict the productivity of vegetation (Hovstad & Ohlson, 2008; Loydi et al., 2013; Ruprecht & Szabó, 2012). Therefore, more photosynthetic products might be distributed to the root to restore the growth of above‐ground plant. Thus, most trace elements in soil could be absorbed by root. The decomposition rate of litter was strongly dependent on the Mn content, because Mn is a key component of lignin decomposing enzymes at the late stage of decomposition (Berg et al., 2007; Tao et al., 2018; Trum et al., 2015; Whalen et al., 2018). The higher the content of Mn, the faster the decomposition of litter (Keiluweit et al., 2015). The Mn content (112.46 mg/kg increased to 138.41 mg/kg) with 39‐year grazing exclusion was higher than that with 18‐year grazing exclusion. This explored that grazing exclusion for 39 years promoted the decomposition of first layer litter. This further explored that over grazing exclusion for 39 year decreased the proportions of trace elements in litter, while made more trace elements distributed in root.

### 4.3 Ecological significance as affected by long‐term grazing exclusion

The deficiency or toxicity of trace elements limits the productivity of plants, and results in enormous economic losses to the herders (Xin et al., 2011; Yadav, 2010). The mean range for Mn content in grass was 71–127 mg/kg (Kabata‐Pendias & Mukherjee, 2007). The Mn content in green plant was decreased from 76.06 mg/kg to 28.14–37.80 mg/kg with 18‐ and 39‐year grazing exclusion. This might cause the deficiency of Mn. The Fe content in the plant was 25–500 mg/kg (Hennessy et al., 2020). The Fe content in the green plant was decreased from 1300 mg/kg to 180–200 mg/kg with 18‐ and 39‐year grazing exclusion. This explored that Fe toxicity occurred in grassland with long‐term grazing, and the deficiency of Mn occurred in grassland with long‐term grazing exclusion. The B content in green plant was changed from 6.62 to 3.89–7.94 mg/kg in grassland with 18‐ and 39‐year grazing exclusion. This was insufficient to meet ruminant dietary requirement of 35–40 mg/kg (Suttle, 2009). In addition, the B stock in green plant was greatly increased with 39‐year grazing exclusion because of a great increase of B content compared to 18‐year grazing exclusion. This indicated that the effective of B resorption in the plant played an important role in the change of B content rather than the dilution effect of long‐term grazing exclusion. B can be transferred from mature or senescent leaves to young organs for reuse when B content in soil is low (Liu et al., 2011). Therefore, lots of B accumulated in green plant and less of B in litter. Less content and stock of B in the root with 39‐year grazing exclusion were observed relative to 18‐year grazing exclusion. This indicated that B was rapidly shifted from the root to the green plant because of the high mobility in phloem and reuse.

## 5 CONCLUSION

The contents and stocks of trace elements in green plant, litter, root, and soil in grassland with long‐term grazing exclusion were investigated for the first time in Inner Mongolia. The Fe toxicity occurred in the grassland with long‐term grazing, and Mn deficiency was observed in the grassland with long‐term grazing exclusion. We confirmed our hypothesis that the distributions of trace elements in grassland system were altered with different years of long‐term grazing exclusion. The trace elements were mainly shifted from the green plant and root to soil and litter with 18‐year grazing exclusion. However, the trace elements were mainly shifted from soil and third layer litter (fragmented litter) to root with continue 39‐year grazing exclusion compared to grazing exclusion for 18 years. The fragmented litter played an important role in the relationship of trace elements between litter and soil. Moreover, the results found that over long‐term grazing exclusion caused the accumulation of trace elements in the root.

## AUTHOR CONTRIBUTIONS

**Juan Hu:** Investigation (equal); writing – original draft (equal). **Daowei Zhou:** Conceptualization (equal).

## CONFLICT OF INTEREST STATEMENT

The authors declare that they have no known competing financial interests or personal relationships that could have appeared to influence the work reported in this paper.

## Data Availability

I confrim that the Data availability statement is included in the main file of my submission, and that access to all necessary data files is provided to editors and reviewers.

## References

[ece370072-bib-0001] Ågren, G. I. (2008). Stoichiometry and nutrition of plant growth in natural communities. Annual Review of Ecology, Evolution, and Systematics, 39(1), 153–170. 10.1146/annurev.ecolsys.39.110707.173515

[ece370072-bib-0002] Alberti, J. , Bakker, E. S. , van Klink, R. , Olff, H. , & Smit, C. (2017). Herbivore exclusion promotes a more stochastic plant community assembly in a natural grassland. Ecology, 98(4), 961–970. 10.1002/ecy.1741/suppinfo 28112395

[ece370072-bib-0003] Bai, W. , Fang, Y. , & Zhou, M. L. (2015). Heavily intensified grazing reduces root production in an Inner Mongolia temperate steppe. Agriculture, Ecosystems and Environment, 200, 143–150. 10.1016/j.agee.2014.11.015

[ece370072-bib-0004] Bai, X. , Yang, X. , Zhang, S. , & An, S. (2021). Newly assimilated carbon allocation in grassland communities under different grazing enclosure times. Biology and Fertility of Soils, 57(4), 563–574. 10.1007/s00374-021-01549-1

[ece370072-bib-0005] Ball, A. B. , Carrillo, Y. , & Molina, M. (2014). The influence of litter composition across the litter–soil interface on mass loss, nitrogen dynamics and the decomposer community. Soil Biology and Biochemistry, 69, 71–82. 10.1016/j.soilbio.2013.10.048

[ece370072-bib-0006] Berg, B. , Steffen, T. K. , & Mcclaugherty, C. (2007). Litter decomposition rate is dependent on litter Mn concentrations. Biogeochemistry, 82(1), 29–39. 10.1007/s10533-006-9050-6

[ece370072-bib-0007] Bowman, W. D. , Cleveland, C. C. , Hreko, U. , Juraj, H. , & Baron, J. S. (2008). Negative impact of nitrogen deposition on soil buffering capacity. Nature Geoscience, 1, 767–770. 10.1038/ngeo339

[ece370072-bib-0008] Chen, Y. , Lee, P. , Lee, G. , & Oikawa, T. (2006). Simulating root responses to grazing of a Mongolian grassland ecosystem. Plant Ecology, 183(2), 265–275. 10.1007/s11258-005-9038-7

[ece370072-bib-0009] Cheng, J. , Jing, G. , Wei, L. , & Jing, Z. (2016). Long‐term grazing exclusion effects on vegetation characteristics, soil properties and bacterial communities in the semi‐arid grasslands of China. Ecological Engineering, 97, 170–178. 10.1016/j.ecoleng.2016.09.003

[ece370072-bib-0010] Congio, G. , Bannink, A. , Mogollón, O. , & Hristov, L. (2021). Enteric methane mitigation strategies for ruminant livestock systems in the Latin America and Caribbean region: A meta‐analysis. Journal of Cleaner Production, 312(S1), 127693. 10.1016/j.jclepro.2021.127693

[ece370072-bib-0011] Dai, L. , Fu, R. , Guo, X. , Du, Y. , & Cao, G. (2021). Long‐term grazing exclusion greatly improve carbon and nitrogen store in an alpine meadow on the northern Qinghai‐Tibet plateau. Catena, 197, 104955. 10.1016/j.catena.2020.104955

[ece370072-bib-0012] Deng, Z. , Shang, Z. N. , & Guan, Z. P. (2014). Long‐term fencing effects on plant diversity and soil properties in China. Soil and Tillage Research, 137, 7–15. 10.1016/j.still.2013.11.002

[ece370072-bib-0013] Deutsch, E. S. , Bork, E. W. , & Willms, W. D. (2010). Soil moisture and plant growth responses to litter and defoliation impacts in parkland grasslands. Agriculture, Ecosystems and Environment, 135(1–2), 1–9. 10.1016/j.agee.2009.08.002

[ece370072-bib-0014] Gao, Y. Z. , Giese, M. , & Lin, S. H. (2008). Belowground net primary productivity and biomass allocation of a grassland in Inner Mongolia is affected by grazing intensity. Plant and Soil, 307(1–2), 41–50. 10.1016/10.1007/s11104-008-9579-3

[ece370072-bib-0015] García, P. , Maestre, T. F. , Kattge, J. H. , & Wall, D. H. (2013). Climate and litter quality differently modulate the effects of soil fauna on litter decomposition across biomes. Ecology Letters, 16(8), 1045–1053. 10.1111/ele.12137 23763716 PMC4411175

[ece370072-bib-0016] Golodets, C. , Kigel, J. , & Sternberg, M. (2010). Recovery of plant species composition and ecosystem function after cessation of grazing in a Mediterranean grassland. Plant and Soil, 329(S1–2), 365–378. 10.1146/10.1007/s11104-009-0164-1

[ece370072-bib-0017] Hafner, S. , Unteregelsbacher, S. , Seeber, E. , Lena, B. , Xu, X. , Li, X. , & Kuzyakov, Y. (2012). Effect of grazing on carbon stocks and assimilate partitioning in a Tibetan montane pasture revealed by ^13^CO_2_ pulse labeling. Global Change Biology, 18(2), 528–538. 10.1111/j.1365-2486.2011.02557.x

[ece370072-bib-0018] Han, W. , Fang, J. Y. , Reich, P. B. , Woodward, F. I. , & Wang, Z. H. (2011). Biogeography and variability of eleven mineral elements in plant leaves across gradients of climate, soil and plant functional type in China. Ecology Letters, 14, 788–796. 10.1111/j.1461-0248.2011.01641.x 21692962

[ece370072-bib-0019] Hansch, R. , & Mendel, R. R. (2009). Physiological functions of mineral micronutrients (Cu, Zn, Mn, Fe, Ni, Mo, B, Cl). Current Opinion in Plant Biology, 12(3), 259–266. 10.1016/j.pbi.2009.05.006 19524482

[ece370072-bib-0020] Hennessy, D. , Delaby, L. , & Dasselaar, V. P. L. (2020). Increasing grazing in dairy cow Milk production Systems in Europe. Sustainability, 12(6), 2443. 10.3390/su12062443

[ece370072-bib-0021] Hoffmann, C. , Funk, R. , & Yong, L. M. (2008). Effect of grazing on wind driven carbon and nitrogen ratios in the grasslands of Inner Mongolia. Catena, 75(2), 182–190. 10.1016/j.catena.2008.06.003

[ece370072-bib-0022] Hou, D. J. , He, W. M. , Liu, C. C. , Qiao, X. G. , & Guo, K. (2019). Litter accumulation alters the abiotic environment and drives community successional changes in two fenced grasslands in Inner Mongolia. Ecology and Evolution, 9(16), 9214–9224. 10.1002/ece3.5469 31463017 PMC6706195

[ece370072-bib-0023] Hovstad, K. A. , & Ohlson, M. (2008). Physical and chemical effects of litter on plant establishment in semi‐natural grasslands. Plant Ecology, 196(2), 251–260. 10.1002/10.1007/s11258-007-9349-y

[ece370072-bib-0024] Hu, J. , Zhou, D. W. , Li, Q. , & Wang, Q. C. (2020). Vertical distributions of soil nutrients and their stoichiometric ratios as affected by Long term grazing and enclosing in a semi‐arid grassland of Inner Mongolia. Agriculture, 10(9), 1–13. 10.3390/agriculture10090382

[ece370072-bib-0025] Hu, J. , Zhou, D. W. , Li, Q. , & Wang, Q. C. (2021). Effects of long‐term enclosing on vertical distributions of soil physical properties and nutrient stocks in grassland of Inner Mongolia. Agronomy, 11, 1832. 10.3390/agronomy11091832

[ece370072-bib-0026] Hu, Z. , Li, S. , Guo, Q. , Niu, S. , He, N. , Li, L. , & Yu, G. (2016). A synthesis of the effect of grazing exclusion on carbon dynamics in grasslands in China. Global Change Biology, 22(4), 1385–1393. 10.1111/gcb.13133 26485056

[ece370072-bib-0027] Javier, Á. , Amelia, G. , & Lasanta, T. (2016). The use of goats grazing to restore pastures invaded by shrubs and avoid desertification: A preliminary case study in the Spanish Cantabrian Mountains. Land Degradation and Development, 27(1), 3–13. 10.1002/ldr.2230

[ece370072-bib-0028] Kabata‐Pendias, A. , & Mukherjee, A. (2007). Trace elements from soil to human: Trace elements from soil to human. Springer Science & Business Media. 10.1007/978-3-540-32714-1

[ece370072-bib-0029] Kang, L. , Han, X. , Zhang, Z. , & Sun, O. J. (2007). Grassland ecosystems in China: Review of current knowledge and research advancement. Philosophical Transactions of the Royal Society of London, 362(1482), 997–1008. 10.1098/rstb.2007.2029 17317645 PMC2435566

[ece370072-bib-0030] Keiluweit, M. , Nico, P. , Harmon, M. E. , Mao, J. , Pett‐Ridge, J. , & Kleber, M. (2015). Long‐term litter decomposition controlled by manganese redox cycling. Proceedings of the National Academy of Sciences of the United States of America, 112, E5253–E5260. 10.1073/pnas.1508945112 26372954 PMC4586821

[ece370072-bib-0031] Li, Y. , Zhao, H. , Zhao, X. , Zhang, T. , Li, Y. , & Cui, J. (2011). Effects of grazing and livestock exclusion on soil physical and chemical properties in desertified sandy grassland, Inner Mongolia, northern China. Environmental Earth Sciences, 63(4), 771–783. 10.1073/10.1007/s12665-010-0748-3

[ece370072-bib-0032] Lin, L. , Li, Y. K. , Xu, X. L. , Zhang, F. W. , Du, Y. G. , Liu, S. L. , & Cao, G. M. (2015). Predicting parameters of degradation succession processes of Tibetan Kobresia grasslands. Solid Earth, 6, 1–10. 10.5194/se-6-1237-2015

[ece370072-bib-0033] Liu, G. D. , Jiang, C. C. , & Wang, Y. H. (2011). Distribution of boron and its forms in young Newhall navel orange (*Citrus sinensis* Osb.) plants grafted on two rootstocks in response to deficient and excessive boron. Soil Science and Plant Nutrition, 57(1), 93–104. 10.1080/00380768.2010.551299

[ece370072-bib-0034] Liu, J. , Li, L. , Chen, X. , Lu, Y. , & Wang, D. (2019). Effects of heat stress on body temperature, milk production, and reproduction in dairy cows: A novel idea for monitoring and evaluation of heat stress‐a review. Asian‐Australasian Journal of Animal Sciences, 32(9), 1332–1339. 10.5713/ajas.18.0743 30744345 PMC6722315

[ece370072-bib-0035] Liu, J. , Wu, J. , Su, H. , Gao, Z. , & Wu, Z. (2017). Effects of grazing exclusion in Xilin Gol grassland differ between regions. Ecological Engineering, 99, 271–281. 10.1016/j.ecoleng.2016.11.041

[ece370072-bib-0036] Liu, M. , Liu, G. H. , & Wu, X. L. (2014). Vegetation traits and soil properties in response to utilization patterns of grassland in Hulun Buir City, Inner Mongolia, China. Chinese Geographical Science, 24(4), 471–478. 10.1007/s11769-014-0706-1

[ece370072-bib-0037] Loydi, A. , Eckstein, L. , Otte, R. , & Annette, W. T. (2013). Effects of litter on seedling establishment in natural and semi‐natural grasslands: A meta‐analysis. Journal of Ecology, 101, 454–464. 10.1111/1365-2745.12033

[ece370072-bib-0038] Mekuria, W. , Veldkamp, E. , Haile, M. , Nyssen, J. , & Gebrehiwot, K. (2007). Effectiveness of exclosures to restore degraded soils as a result of overgrazing in Tigray, Ethiopia. Journal of Arid Environments, 69(2), 270–284. 10.1016/j.jaridenv.2006.10.009

[ece370072-bib-0040] Mills, A. J. , Strydom, T. , Allen, J. L. , & Baum, J. (2023). Potential geochemical constraints on tree seedlings in northern Kruger National Park grasslands. Koedoe: African Protected Area Conservation and Science, 65, 1773. 10.4102/koedoe.v65i1.1773

[ece370072-bib-0041] Naeem, I. , Wu, X. F. , & Asif, T. D. (2021). Livestock diversification implicitly affects litter decomposition depending on altered soil properties and plant litter quality in a meadow steppe. Plant and Soil, 473, 49–62. 10.1007/s11104-021-05006-8

[ece370072-bib-0042] Ning, J. , Liu, S. S. , Chang, S. H. , Chen, X. J. , West, C. P. , & Hou, F. J. (2022). Dominant species as biological indicators to predict the changes of trace element in different types of rangeland. Ecological Indicators, 137, 108735. 10.1016/j.ecolind.2022.108735

[ece370072-bib-0043] Oliveira Filho, J. D. S. , Vieira, J. N. , Ribeiro da Silva, E. M. , Beserra de Oliveira, J. G. , Pereira, M. G. , & Brasileiro, F. G. (2019). Assessing the effects of 17 years of grazing exclusion in degraded semi‐arid soils: Evaluation of soil fertility, nutrients pools and stoichiometry. Journal of Arid Environments, 166, 1–10. 10.1016/j.jaridenv.2019.03.006

[ece370072-bib-0044] Pei, S. , Hua, F. , & Wan, C. (2008). Changes in soil properties and vegetation following exclosure and grazing in degraded Alxa desert steppe of Inner Mongolia, China. Agriculture, Ecosystems and Environment, 124(1), 33–39. 10.1016/j.agee.2007.08.008

[ece370072-bib-0045] Piñeiro, G. , Paruelo, J. M. , & Jobbágy, E. G. (2009). Grazing effects on belowground C and N stocks along a network of cattle exclosures in temperate and subtropical grasslands of South America. Global Biogeochemical Cycles, 23, GB2003. 10.1029/2007GB003168

[ece370072-bib-0046] Qasim, S. , Gul, S. , Shah, M. H. , Hussain, F. , Ahmad, S. , Islam, M. , & Shah, S. Q. (2017). Influence of grazing exclosure on vegetation biomass and soil quality. International Soil and Water Conservation Research, 5(1), 62–68. 10.1016/j.iswcr.2017.01.004

[ece370072-bib-0047] Ren, Y. , Lü, Y. , & Fu, B. (2016). Quantifying the impacts of grassland restoration on biodiversity and ecosystem services in China: A meta‐analysis. Ecological Engineering, 95, 542–550. 10.1016/j.ecoleng.2016.06.082

[ece370072-bib-0048] Roy, S. , Naidu, D. G. T. , & Bagchi, S. (2023). Functional substitutability of native herbivores by livestock for soil carbon stock is mediated by microbial decomposers. Global Change Biology, 29, 2141–2155. 10.1111/gcb.16600 36732877

[ece370072-bib-0049] Ruprecht, E. , & Szabó, A. (2012). Grass litter is a natural seed trap in long‐term undisturbed grassland. Journal of Vegetation Science, 23(3), 495–504. 10.1111/j.1654-1103.2011.01376.x

[ece370072-bib-0051] Shen, X. Y. , Du, G. Z. , & Hong, L. (2006). Studies of a naturally occurring molybdenum‐induced copper deficiency in the yak. Veterinary Journal, 171(2), 352–357. 10.1016/j.tvjl.2004.11.006 16490720

[ece370072-bib-0052] Shrestha, G. , & Stahl, P. D. (2008). Carbon accumulation and storage in semi‐arid sagebrush steppe: Effects of long‐term grazing exclusion. Agriculture, Ecosystems and Environment, 125(1), 173–181. 10.1016/j.agee.2007.12.007

[ece370072-bib-0053] Song, X. , Wang, L. , Zhao, X. , Liu, C. , Chang, Q. , Wang, Y. , & Wang, D. (2017). Sheep grazing and local community diversity interact to control litter decomposition of dominant species in grassland ecosystem. Soil Biology and Biochemistry, 115, 364–370. 10.1016/j.soilbio.2017.09.003

[ece370072-bib-0054] Steffens, M. , Kolbl, A. , & Totsche, K. U. I. (2008). Grazing effects on soil chemical and physical properties in a semiarid steppe of Inner Mongolia (P.R. China). Geoderma, 143(1–2), 63–72. 10.1016/j.geoderma.2007.09.004

[ece370072-bib-0055] Stromberg, C. A. E. , & Staver, A. C. (2022). The history and challenge of grassy biomes grassy biomes are >20 million years old but are undervalued and under threat today. Science, 377, 592–593. 10.1126/science.add1347 35926015

[ece370072-bib-0056] Suttle, N. F. (2009). Mineral nutrition of livestock (Vol. 215, pp. 1–8). Cabi Bookshop.

[ece370072-bib-0057] Tao, S. , Cui, Y. , & Berg, B. L. (2018). A test of manganese effects on decomposition in forest and cropland sites. Soil Biology and Biochemistry, 129, 178–183. 10.1016/j.soilbio.2018.11.018

[ece370072-bib-0058] Trum, F. , Titeux, H. , & Ponette, Q. B. (2015). Influence of manganese on decomposition of common beech (*Fagus sylvatica* L.) leaf litter during field incubation. Biogeochemistry, 15(3), 1–10. 10.1007/s10533-015-0129-9

[ece370072-bib-0059] Wang, J. , Zhao, M. L. , Willms, W. D. , & Han, Y. F. (2011). Can plant litter affect net primary production of a typical steppe in Inner Mongolia? Journal of Vegetation Science, 22(2), 367–376. 10.1111/j.1654-1103.2011.01257.x 32336913 PMC7166792

[ece370072-bib-0060] Wang, L. , Wiesmeier, M. , Zhao, G. , Zhang, R. , Hou, F. , Han, G. , & Gan, Y. (2018). Grazing exclusion—An effective approach for naturally restoring degraded grasslands in northern China. Land Degradation and Development, 29(12), 4439–4456. 10.1002/ldr.3191

[ece370072-bib-0061] Wang, Z. , Deng, X. , Song, W. , Li, Z. , & Chen, J. (2017). What is the main cause of grassland degradation? A case study of grassland ecosystem service in the middle‐south Inner Mongolia. Catena, 150, 100–107. 10.1016/j.catena.2016.11.014

[ece370072-bib-0062] Whalen, D. E. , Smith, G. R. , & Grandy, S. A. D. S. (2018). Manganese limitation as a mechanism for reduced decomposition in soils under atmospheric nitrogen deposition. Soil Biology and Biochemistry, 127, 252–263. 10.1016/j.soilbio.2018.09.025

[ece370072-bib-0064] Wu, G. L. , Liu, Z. H. , Lei, Z. , Chen, J. M. , & Hu, T. M. (2010). Long‐term fencing improved soil properties and soil organic carbon storage in an alpine swamp meadow of western China. Plant and Soil, 332(S1–2), 331–337. 10.1007/s11104-010-0299-0

[ece370072-bib-0065] Xin, G. S. , Long, R. J. , Guo, X. S. , Irvine, J. , Ding, L. M. , Ding, L. L. , & Shang, Z. H. (2011). Blood mineral status of grazing Tibetan sheep in the northeast of the Qinghai–Tibetan plateau. Livestock Science, 136(2–3), 102–107. 10.1016/j.livsci.2010.08.007

[ece370072-bib-0066] Yadav, S. K. (2010). Heavy metals toxicity in plants: An overview on the role of glutathione and phytochelatins in heavy metal stress tolerance of plants. South African Journal of Botany, 76(2), 167–179. 10.1016/j.sajb.2009.10.007

[ece370072-bib-0067] Yan, L. , Han, G. , He, Z. , Zhao, M. , Snyman, H. A. , & Dan, S. (2009). Grazing intensity on vegetation dynamics of a typical steppe in Northeast Inner Mongolia. Rangeland Ecology & Management, 62(4), 328–336. 10.2111/08-167.1

[ece370072-bib-0068] Zeng, Q. , An, S. , & Yang, L. (2017). Soil bacterial community response to vegetation succession after fencing in the grassland of China. Science of the Total Environment, 609(31), 2–10. 10.1016/j.scitotenv.2017.07.102 28732294

[ece370072-bib-0069] Zhang, P. , Li, B. , Wu, J. H. , & Shui, J. (2019). Invasive plants differentially affect soil biota through litter and rhizosphere pathways: A meta‐analysis. Ecology Letters, 22(1), 200–210. 10.1111/ele.13181 30460738

[ece370072-bib-0070] Zhao, L. H. , Li, S. G. , Zhang, H. T. , & Zhou, O. (2004). Sheep gain and species diversity: In Sandy grassland, Inner Mongolia. Journal of Range Management, 57(2), 187–190. 10.2307/4003917

[ece370072-bib-0072] Zhou, Z. Y. , Li, F. R. , Chen, S. K. , Zhang, H. R. , & Li, G. (2011). Dynamics of vegetation and soil carbon and nitrogen accumulation over 26 years under controlled grazing in a desert shrubland. Plant and Soil, 341(1–2), 257–268. 10.1007/s11104-010-0641-6

